# The dual role of p62 in ferroptosis of glioblastoma according to p53 status

**DOI:** 10.1186/s13578-022-00764-z

**Published:** 2022-02-25

**Authors:** Fanen Yuan, Qian Sun, Si Zhang, Liguo Ye, Yang Xu, Gang Deng, Zhou Xu, Shenqi Zhang, Baohui Liu, Qianxue Chen

**Affiliations:** 1 Department of Neurosurgery, Renmin Hospital of Wuhan University, Wuhan, Hubei 430060 People’s Republic of China; 2 Central Laboratory, Renmin Hospital of Wuhan University, Wuhan, Hubei 430060 People’s Republic of China

**Keywords:** Glioblastoma, Ferroptosis, p62, p53, NRF2

## Abstract

**Background:**

Ferroptosis plays a key role in human cancer, but its function and mechanism in glioma is not clear. P62/SQSTM1 was reported to inhibit ferroptosis via the activation of NRF2 signaling pathway. In this study we reveal a dual role of p62 in ferroptosis of glioblastoma (GBM) according to p53 status.

**Method:**

Lipid peroxidation analysis, transmission electron microscopy (TEM), GSH assay were performed to determine the level of ferroptosis. Western blot and qPCR were obtained to detect the expression of ferroptosis markers. Construction of mutant plasmids, immunoprecipitation, luciferase assay and rescue-experiments were performed to explore the regulatory mechanism.

**Results:**

P62 overexpression facilitates ferroptosis and inhibits SLC7A11 expression in p53 mutant GBM, while attenuates ferroptosis and promotes SLC7A11 expression in p53 wild-type GBM. P62 associates with p53 and inhibits its ubiquitination. The p53-NRF2 association and p53-mediated suppression of NRF2 antioxidant activity are diversely regulated by p62 according to p53 status. P53 mutation status is required for the dual regulation of p62 on ferroptosis. In wild-type p53 GBM, the classical p62-mediated NRF2 activation pathway plays a major regulatory role of ferroptosis, leading to increased SLC7A11 expression, resulting in a anti-ferroptosis role. In mutant p53 GBM, stronger interaction of mutant-p53/NRF2 by p62 enhance the inhibitory effect of mutant p53 on NRF2 signaling, which reversing the classical p62-mediated NRF2 activation pathway, together with increased p53’s transcriptional suppression on SLC7A11 by p62, leading to a decrease of SLC7A11, resulting in a pro-ferroptosis role.

**Conclusion:**

Together, this study shows novel molecular mechanisms of ferroptosis regulated by p62; the mutation status of p53 is an important factor that determines the therapeutic response to p62-mediated ferroptosis-targeted therapies in GBM.

**Supplementary Information:**

The online version contains supplementary material available at 10.1186/s13578-022-00764-z.

## Introduction

Glioma is the most common primary malignant tumor in adult central nervous system. Glioblastoma (GBM) is the most malignant type of gliomas. GBM is characterized with high degree of malignancy, high recurrence rate and poor prognosis. The median survival period of GBM was only 14.6 months, and the 5-year survival rate was only 4–5% [1, 2]. To develop the effective therapies, the comprehensive understanding of the molecular mechanism in GBM is urgently required.

Ferroptosis is a recently discovered form of programmed cell death driven by the generation of lipid-based reactive oxygen species (ROS) tightly linked with oxidative stress responses and cystine metabolism [3, 4]. Accumulating evidence indicates that ferroptosis plays an important role in human cancers, including gliomas [5–7]. Ferroptosis inducers such as erastin and RSL3 can promote tumor cell death and inhibit the growth of tumor cells alone or in combination with chemotherapy drugs [5]. It is reported that erastin sensitizes glioblastoma cells to temozolomide by restraining xCT [6]. Stockwell et al. found that radiation induced lipid oxidation can promote ferroptosis, and ferroptosis inducer can enhance the killing effect of radiotherapy on lung adenocarcinoma and glioma [7]. Targeted therapy for ferroptosis may be an effective supplement to the comprehensive treatment of GBM.

P62/SQSTM1 (hereafter as p62), a stress-inducible adapter protein, has been widely identified to function as a receptor for ubiquitinated proteins [8]. NRF2 is a key factor responsible for protecting against oxidative damage by the transcriptional activation of antioxident genes. P62 interacts with Keap1 (a negative regulator of NRF2), releases NRF2 from the inhibition of Keap1, resulting in an activation of NRF2 [9]. Recent evidence reported that the p62-NRF2 pathway plays a important role in ferroptosis [10]. However, the role and exact mechanism of p62-NRF2 pathway in the regulation of GBM ferroptosis is not clear.

More than 50% of human tumors acquire a mutation in p53. It is generally believed that p53 plays a key role in the regulation of ferroptosis. Under different conditions, p53 may enhance or inhibit ferroptosis [11–13]. Moreover, accumulating evidence indicates that p53 regulates NRF2 signaling and mutant p53 may interact with NRF2 differentially regulating the NRF2-mediated antioxidant response [14–16]. The crosstalk between p62, NRF2 and p53 in ferroptosis remains unclear, and its association with p53 status requires further studies.

Different from p53 wild-type GBM cell lines [U87 (p53 WT), A172 (p53 WT)], U251 (p53 R273H) and U118 (p53 R213Q) contain different p53 mutations [17]. Marie et al. confirmed p53 R273H mutation in U251 cells by sequencing of PCR-amplified p53 cDNA, and established a small library of genomic sequences bound by p53 (R273H) in U251 using ChIP [18]; they provided evidence that p53 R273H protein is targeted to the nucleus of U251 cells where it interacts with DNA and participates in the regulation of gene transcription. By querying on ATCC website, U118 is one of a number of cell lines derived from malignant gliomas by J. Ponten and associates from 1966 to 1969 [19].

In this study we reveal a dual role of p62 in ferroptosis of glioblastoma according to p53 status. P62 overexpression facilitates ferroptosis and inhibits SLC7A11 expression in p53 mutant GBM, while attenuates ferroptosis and promotes SLC7A11 expression in p53 wild-type GBM. P62 associates with p53 and inhibits its ubiquitination. The p53-NRF2 association and p53-mediated suppression of NRF2 antioxidant activity are diversely regulated by p62 according to p53 status. P53 mutation status is required for the dual regulation of p62 on ferroptosis. It is speculated that the classical p62-mediated NRF2 activation pathway plays the major regulatory role in p53-WT GBM, thus resulting in the suppression of ferroptosis; while p62-mediated p53-SLC7A11 axis plays the major regulatory role in p53-mutant GBM, consistent with stronger mutant-p53-NRF2 association mediated by p62 and enhanced inhibition of NRF2 antioxidant activity, leading to the induction of ferroptosis.

## Material and methods

### Antibodies and reagents

The antibodies included the following: anti-SQSTM1/p62 (M162-3, Medical Biological Laboratories, Japan), anti-SQSTM1/p62 (ab109012, Abcam, USA), anti-p53 (sc-126, Santa Cruze Biotechnology, USA), anti-p53 (10,442–1-AP, Proteintech, China), anti-mutant p53 (ab32049, Abcam), anti-NRF2 (M200-3, Medical Biological Laboratories), anti-NRF2 (16,396–1-AP, Proteintech), anti-ubiquitin (10,201–2-AP, Proteintech), anti-GAPDH (#5174, Cell Signaling Technology), anti-β-actin (#4970, Cell Signaling Technology), anti-HA (M180-3 and M561, Medical Biological Laboratories), anti-His (D291-3, Medical Biological Laboratories), anti-Flag (M185, Medical Biological Laboratories), anti-Flag (20,543–1-AP, Proteintech), anti-SLC7A11 (NB300-318, Novus, USA), anti-SLC7A11 (26,864–1-AP, Proteintech) anti-HO1 (ab68477, Abcam), anti-HO1 (10,701–1-AP, Proteintech), anti-NQO1 (ab80588, Abcam), anti-Keap1 (10,503–2-AP, Proteintech).

The reagents included the following: Erastin (S7242, Selleck, USA), APR-246 (HY-19980, MCE, USA), Pifithrin-α (HY-15484, MCE), Nutlin-3 (S1061, Selleck).

### Cell culture and treatment

Human glioblastoma cell lines (U251, U118, U87 and A172) and HEK 293 T cells were obtained from the Cell Bank of the Shanghai Institute of Biochemistry and Cell Biology, Chinese Academy of Sciences (Shanghai, China). Cells were cultured in high-glucose DMEM (Gibco, Thermo Fisher Scientific) containing 10% foetal bovine serum (Thermo Fisher Scientific) and 1% penicillin/streptomycin (Thermo Fisher Scientific). Erastin was used at 10 μM. APR-246 was used at 10 μM. Pifithrin-α was used at 10 μM. Nutlin-3 was used at 10 μM.

### Human tissue samples

Human glioma specimens were collected from the Department of Neurosurgery, Renmin Hospital of Wuhan University, Wuhan, China. Patients were asked to submit written informed consent and were approved by the Institutional Ethics Committee of the Faculty of Medicine at Renmin Hospital of Wuhan University (approval number: 2012LKSZ (010) H). The collected specimen were histologically determined by pathologists at Renmin Hospital of Wuhan University.

### DNA construction and transfection

The HA-p62 plasmids were constructed based on the pcDNA3.1. The HA-p62 mutant fragments (Δ1–385), (Δ124–440), (Δ230–440) and (Δ1–227, 245–442) were generated from HA-p62 wild type. “Δ1–385” means that residues 1–385 are retained and other region residues are removed. HA-p53 (wild type) plasmids, flag-p53 (wild type) plasmids, flag-R273H-p53 (mutant) plasmids and HA-NRF2 plasmids were constructed based on the pcDNA3.1. His-ubiquitin and its indicated mutants (His-K48R-Ub, His-K63R-Ub) were constructed based on the pcDNA3.1. Transfections were performed using Lipofectamine 3000 transfection reagent (L3000015, Thermo Fisher Scientific) according to the manufacturer's instructions.

### CRISPR/CAS9

CRISPR/CAS9-mediated SQSTM1/p62 depletion was applied according to Zhang Feng Lab’s protocol (http://www.addgene.org/crispr/zhang/). Two sgRNA targeting SQSTM1/p62 was designed using CRISPRdirect (http://crispr.dbcls.jp/) and the oligo-sequences for used sgRNAs:

oligo 1 (5′-caccgcgttcgctacaaaagccgcg-3′ and 3′-cgcaagcgatgttttcggcgccaaa-5′).

oligo 2 (5′-caccgcgccagctcgccgctcgcta-3′ and 3′-cgcggtcgagcggcgagcgatcaaa-5′).

The designed sgRNA were cloned into lentiCRISPRv2 vector (#52961, Addgene, USA).The constructed vectors were validated by sequencing. LentiCRISPRv2-p62 or lentiCRISPRv2 was co-transfected with the packaging vectors pSPAX2 (#12260, Addgene) and pMD2.G (#12259, Addgene) into 293 T cells using Lipofectamine 2000 transfection reagent (Thermo Fisher Scientific) to generate lentiviruses. The supernatants containing the lentiviruses were collected and used to infect U251 or U87 cells. Cells were selected with 8 mg/mL puromycin for at least 48 h. Western blot was performed to verify the knockout efficacy.

### Quantitative RT-PCR

Total RNA were isolated by using the trizol reagent (Invitrogen, USA), and cDNA was synthesized using a PrimeScript RT Reagent Kit with gDNA Eraser (RR047A, Takara, Japan) according to manufacturer’s guidelines. For quantitative RT-PCR, SYBR® Premix Ex Taq™ II (RR820A, Takara) and CFX96 Real-Time PCR System (Biorad, USA) were used according to the standard protocols. Expression levels were normalized to GAPDH. The primers are listed as follows:

GAPDH: 5′-GGAGCGAGATCCCTCCAAAAT-3′ (Forward),

5′-GGCTGTTGTCATACTTCTCATGG-3′ (Reverse).

SQSTM1/p62: 5′-GCACCCCAATGTGATCTGC-3′ (Forward),

5′-CGCTACACAAGTCGTAGTCTGG-3′ (Reverse).

SLC7A11: 5′-TCTCCAAAGGAGGTTACCTGC-3′ (Forward),

5′-AGACTCCCCTCAGTAAAGTGAC-3′ (Reverse).

### Immunofluorescence

Cells were grown on coverslips and fixed in 4% paraformaldehyde for 15 min, permeabilized with 1% Triton X-100 in 1 × PBS for 10 min, blocked in 1% bovine serum albumin for 1 h at room temperature. Then cells were incubated with indicated primary antibodies overnight, followed by Alexa fluor-labelled secondary antibody (Antgene, Wuhan, China). Images were captured using Olympus microscope.

### Lipid peroxidation analysis

Analysis of lipid peroxidation was performed by Lipid Peroxidation (MDA) Assay Kit (S0131S, Beyotime, China) following manufacturer’s instructions. MDA concentration was normalized to protein concentrations, which were detected by the BCA method with the kit (#AR0197, Boster Biological &Technology, China).

### Transmission electron microscopy (TEM)

Cells were fixed with a 2.5% glutaraldehyde solution. The cells were then post-fixed in 1% osmic acid. A graded series of ethanol was used to dehydrate the specimens. They were then placed in capsules contained embedding medium and heated at 70 °C for approximately 9 h. The specimen sections were stained by uranyl acetate and alkaline lead citrate. Images were acquired using a TEM (Hitachi HT7700, Japan).

### Immunohistochemistry

Tissue samples embedded in paraffin were sectioned, deparaffinized, and subjected to antigen retrieval performed in citrate buffer (pH 6.0). Peroxidase was blocked using the 3% H2O2 for 10 min. After being blocked with 1% BSA for 1 h, the sections were incubated in primary antibodies overnight, followed by HRP-labelled secondary antibody (Servicebio, China). Signals were detected using DAB staining (Servicebio), and the samples were counterstained with haematoxylin. Images were obtained using an Olympus BX51 microscope (Olympus).

### Measurement of GSH

The relative glutathione (GSH) concentration in cell lysates was determined by the GSH and GSSG Assay Kit (S0053, Beyotime, China) according to the manufacturer’s instructions.

### Western Blot

Total proteins from the cultured cells was extracted using RIPA Lysis Buffer (P0013B, Beyotime, China). BCA assay kit (PC0020, Solarbio, China) was used to determine the protein concentration. Proteins were subjected to SDS-PAGE and transferred onto PVDF membranes. After antigen blocking with 5% non-fat milk, the blots were incubated with primary antibodies overnight at 4 °C, followed by Alex Fluor 680/790-labelled secondary antibodies (LI-COR Bioscience, USA) or HRP-labelled secondary antibodies at room temperature for 1 h. Subsequently, the bands were visualized with LI-COR Odyssey Infrared Imaging System (LI-COR Biosciences) or ChemiDoc™ Touch Imaging System (BIO RAD).

### Immunoprecipitation

Proteins were lysed in IP buffer (2 mM Tris–Cl, pH 8.0, 0.5 mM EDTA, 150 mM NaCl, 1% NP-40, 50 mM NaF, 2 mM Na3VO4, 4 mM Na pyrophosphate, and 25 μL protein inhibitor/mL). The proteins were incubated with primary antibodies or IgG (Beyotime) for 4 h at 4 °C. Subsequently, 30 μL of Protein A/G PLUS-Agarose (sc-2003, Santa Cruz Biotechnology) was added to the mixture and incubated at 4 °C overnight. The immunoprecipitates were washed five times in IP buffer, resuspended in 40 μL of 1.5 × loading buffer, and analyzed by western blot analysis.

### Intracranial xenograft model

Cells were stereotactically implanted into the brain of nude mice under anesthesia as previously described [20]. Mice were monitored periodically and sacrificed when they showed severe neurological symptoms and/or obvious weight loss (> 20% of their body weight). For histological analyses, the whole brains of the mice were removed, fixed in formalin and embedded in paraffin. Animal experiments were performed in accordance with animal care ethics approval and guidelines.

### Luciferase assay

Luciferase assay was performed to detect the activity of NRF2 signaling pathway. ARE luciferase reporter (wild-type NRF2 binding sites) plasmids were constructed by Miaoling Biology (Wuhan, China). The pGMLR-TK luciferase reporter plasmid was purchased from Yeasen Technology (Shanghai, China) and used for renilla luciferase reporter as a control. The dual luciferase assay was performed using Dual Luciferase Reporter Gene Assay Kit (Yeasen Technology, Shanghai, China) according to the manufacturer’s instructions. Luciferase activity was calculated by normalizing the luciferase with the corresponding renilla value and represented as relative luciferase activity.

### Single-sample gene set enrichment analysis (ssGSEA)

In TCGA-glioma dataset, 587 tumor samples were divided into four subtypes, p53-mutant LGG, p53-Wild-type LGG, p53-mutant GBM and p53-Wild-type GBM, according to the status of p53 mutation. In each subtype, samples were assigned to low or high expression group by the median value of p62 or NRF2. Furthermore, the gene set “WP_Ferroptosis” representing the activity of ferroptosis pathways were obtained from Molecular Signatures Database (http://www.broad.mit.edu/gsea/msigdb/), then the gene set was quantified for their enrichment degrees (enrichment score, ES) within respective in each glioma samples using single-sample gene-set enrichment analysis (ssGSEA) by R package “GSVA”. Difference analysis was performed among low and high expression group in each subtype by R package “ggpubr”, p < 0.05 was considered significant.

### Survival analysis

According to p53 mutation status and the median of p62 or NRF2 respectively, patients with glioma were divided into p53 mutant + p62/NRF2 low expression, p53 wild-type + p62/NRF2 low expression, p53 mutant + p62/NRF2 high expression and p53 wild-type + p62/NRF2 high expression group. Kaplan-Maier curve were performed by R package “survival” among four groups in p62 or NRF2 respectively to evaluate the association of overall survival (OS) and four groups.

### Cell death assays

Cells after indicated treatment were trypsinized, and stained with Trypan blue. Viable cells were then counted using a hemocytometer under a microscope. The blue-stained cells were considered dead.

### Statistical analysis

Statistical significance was determined by Student’s t-test. Survival analysis was performed by the Kaplan–Meier method and the log-rank test. Data are expressed as means ± SEM. P values were calculating by Graphpad prism 5.0, and P < 0.05 was considered significant.

## Results

### P62 promotes ferroptosis in p53-mutant GBM cells but inhibits ferroptosis in p53-wild-type GBM cells

**Fig. 1:**
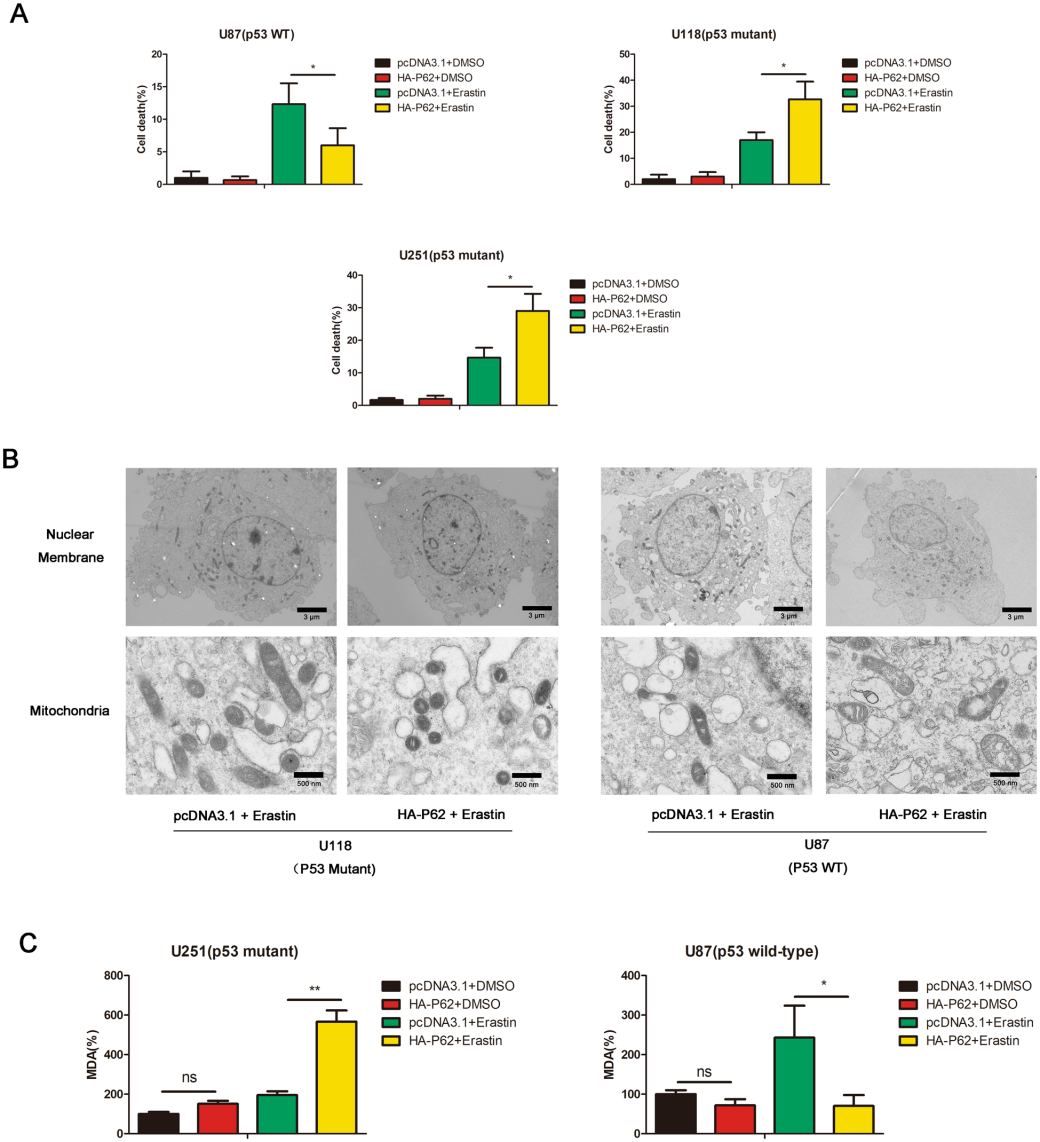
P62 promotes ferroptosis in p53-mutant GBM cells but inhibits ferroptosis in p53-wild-type GBM cells. **A** U118, U251 or U87 cells were transfected with pcDNA3.1 or HA-P62 plasmids, followed by DMSO or erastin treatment. Cell death were determined by trypan blue staining. **B** U118 or U87 control cells and p62-overexpressed clones treated with erastin were subjected to transmission electron microscopy. **C** The levels of MDA were measured in U251 or U87 cells, which were transfected with pcDNA3.1 or HA-P62 plasmids, followed by DMSO or erastin treatment. pcDNA3.1: the control group. HA-P62: p62 overexpression group

The p53 status of GBM cells used in our study is as follows [17, 21]: U251 (p53 R273H), U118 (p53 R213Q), U87 (p53 wild type) and A172 (p53 wild type). As shown in Fig. 1A, high levels of cell death were observed upon erastin induction in p62-overexpressed U118 (p53 mutant) cells and U251 (p53 mutant) cells comparing to its parental control cells. But in U87 (p53 wild-type) cells, p62-overexpressed cells showed low levels of cell death upon erastin induction comparing to its parental control cells (Fig. 1A). Shrunken mitochondria with increased membrane density is considered as characteristic morphologic feature of ferroptotic cells [12]. By transmission electron microscopy, we observed shrunken mitochondria with increased membrane density in p62-overexpressed U118 (p53 mutant) cells, but not in p62-overexpressed U87 (p53 wild-type) cells (Fig. 1B). Lipid peroxidation is an important event in ferroptosis. P62 overexpression significantly resulted in an increase of lipid peroxidation in U251 (p53 mutant) cells, but p62 overexpression resulted in a decrease of lipid peroxidation in U87 (p53 wild-type) cells (Fig. 1C). All these results indicated that p62 promotes ferroptosis in p53-mutant GBM cells but inhibits ferroptosis in p53-wild-type GBM cells.

### P62 negatively regulates SLC7A11 expression in p53-mutant GBM but positively regulates SLC7A11 expression in p53-wild-type GBM cells

SLC7A11, which is involved in the formation of system XC -, is recognized as a key factor of anti-ferroptosis [12, 22] and is regulated by both NRF2 and p53 signaling pathway [12, 22]. Endogenous expression of SLC7A11, p53, p62 and NRF2 were examined by western blot in U251, U118, U87 and A172 cells under normal conditions (Additional file 1: Figure S1).

**Fig. 2:**
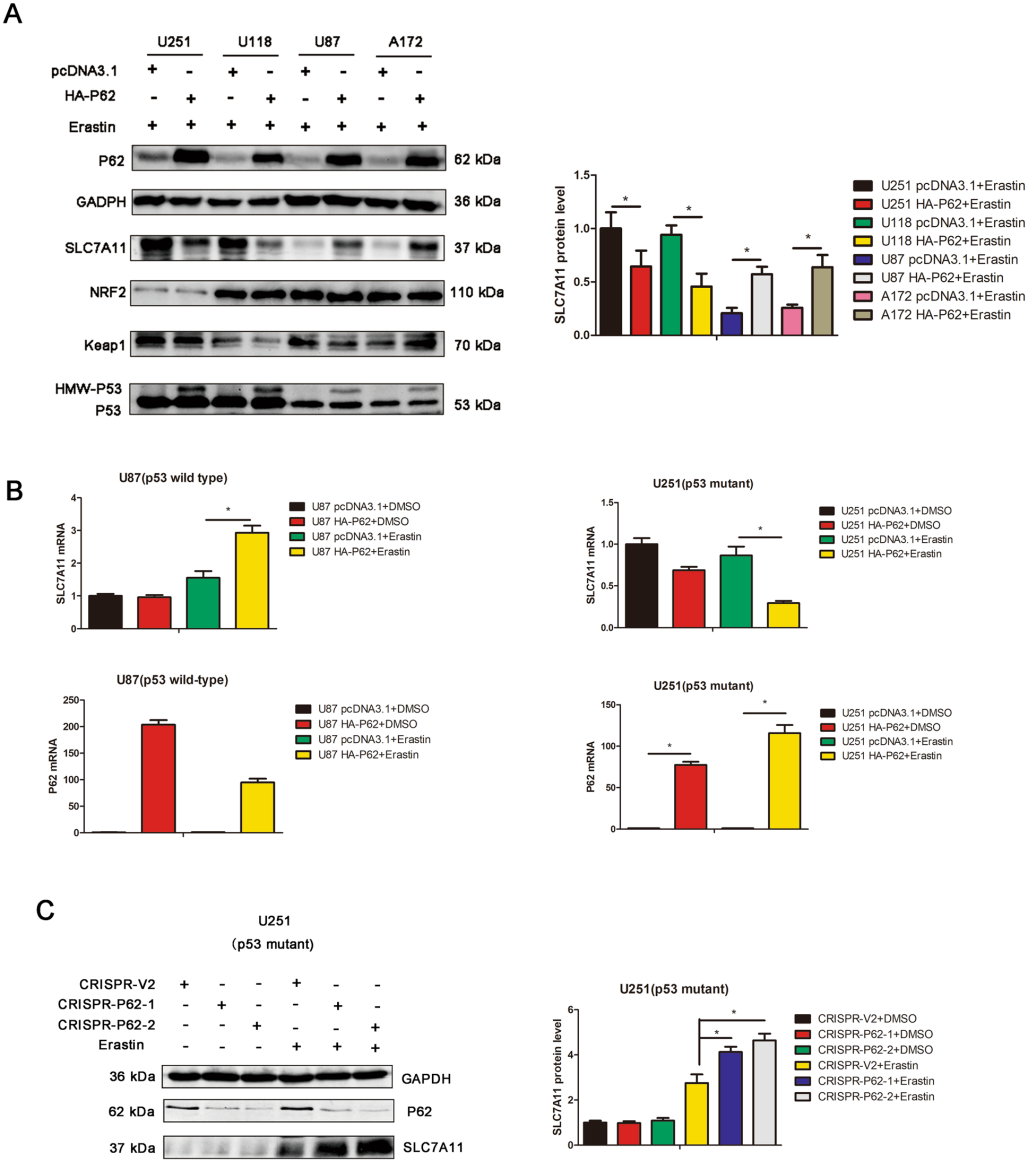
P62 negatively regulates SLC7A11 expression in p53-mutant GBM cells but positively regulates SLC7A11 expression in p53-wild-type GBM cells. **A** U251, U118, U87 and A172 cells were transfected with pcDNA3.1 or HA-P62 plasmids, followed by erastin treatment. The protein expression of SLC7A11, NRF2, P53, Keap1 and P62 were detected by western blot analysis. The quantification of western blots were also presented. **B** U251 and U87 cells were transfected with pcDNA3.1 or HA-P62 plasmids, followed by DMSO or erastin treatment. The mRNA expression of P62 and SLC7A11 were measured by qPCR. mRNA expression was normalized to GAPDH using the 2^−ΔΔCt^ method. **C** CRISPR/CAS9 system was performed to knockout p62 expression in U251 cells. SLC7A11 and p62 expression were detected by western blot analysis

We examined the protein expression of SLC7A11, NRF2, Keap1 and p53 in p62-overexpressed and its parental control cells treated with eastin by western blot. The results indicated that p62 overexpression increased the protein expression of SLC7A11 in U87 (p53 wild-type) and A172 (p53 wild-type) cells but decreased the protein expression of SLC7A11 in U251 (p53 mutant) and U118 (p53 mutant) cells upon erastin treatment (Fig. 2A). Interesting, p62 overexpression resulted in an increase of HMW (high molecular weight)-p53 expression, despite of p53 status (Fig. 2A). The protein expression of NRF2 and Keap1 remains unchanged upon p62 overexpression in U251, U118 and U87 cells (Fig. 2A).

We performed quantitative RT-PCR to examine the mRNA expression of SLC7A11. In U87 (p53 wild-type) cells treated with erastin, p62 overexpression increased the mRNA expression of SLC7A11 (Fig. 2B). But in U251 (p53 mutant) cells treated with erastin, p62 overexpression decreased the mRNA expression of SLC7A11 (Fig. 2B). CRISPR/CAS9 system was conducted to generate p62 knockout clones, and western blot indicated that p62 depletion resulted in an increase of SLC7A11 expression in U251 (p53 mutant) cells treated with erastin (Fig. 2C).

All above results showed that p62 diversely regulates SLC7A11, a key ferroptosis marker, in p53-mutant or in p53-wild-type GBM cells. In p53 wild-type GBM, p62 positively regulates SCL7A11. In p53 mutant GBM, p62 negatively regulates SCL7A11. Notably, SLC7A11 is regulated by p53 and NRF2, and it remind us to explore the effect of p62 on p53 and NRF2 signaling pathway to explain the discrepancy.

### P62 associates with p53 and inhibits the ubiquitination of p53

**Fig. 3:**
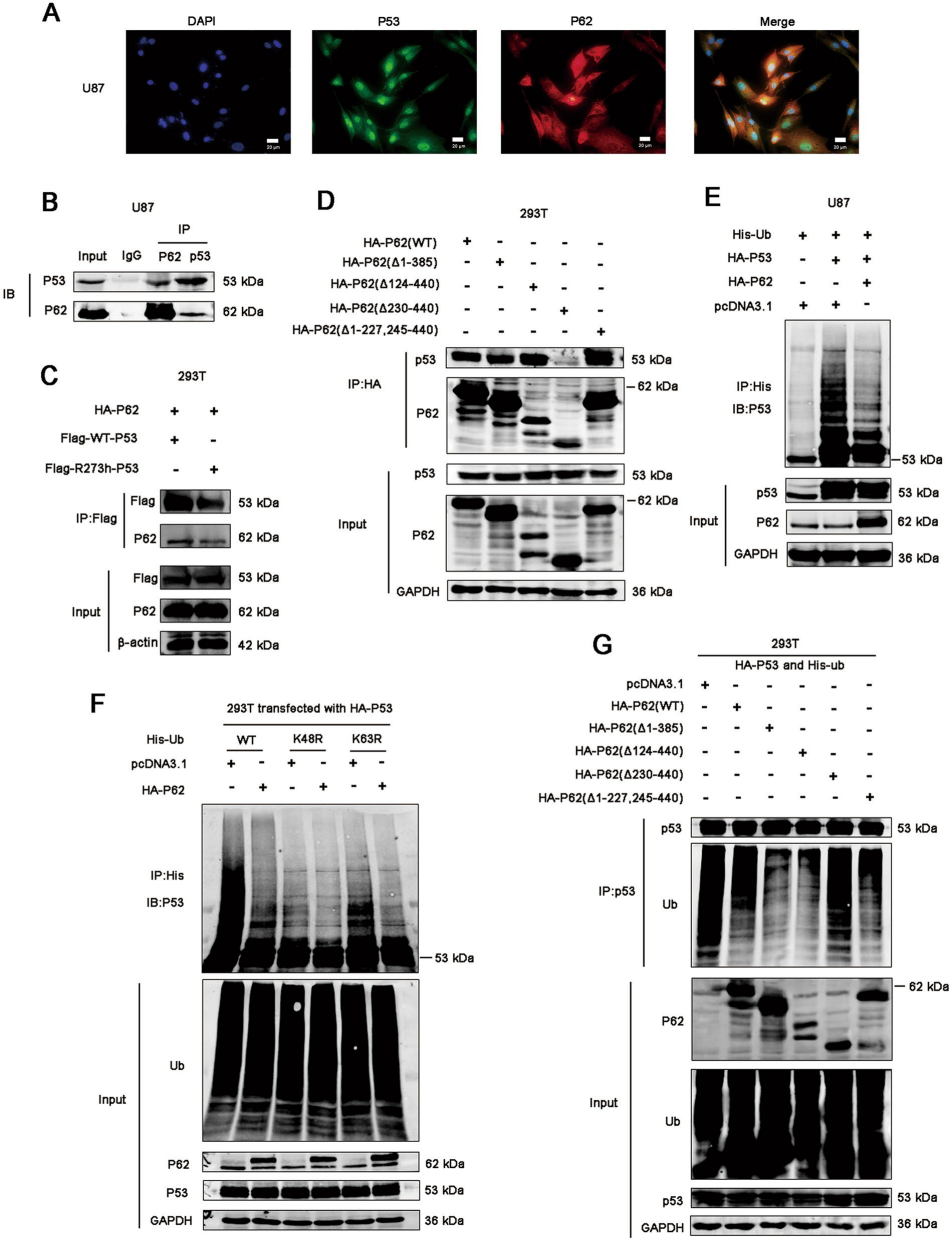
P62 associates with p53 and inhibits its ubiquitination. **A** The localization of p62 and p53 was detected by immunofluorescence in U87 cells. **B** Co-immunoprecipitation assays were performed to assess the association between p62 and p53. Lysates of U87 cells were subjected to IP using anti-P62 or anti-P53 antibodies, followed by immunoblotting with anti-P62 and anti-P53 antibodies. Non-specific IgG was used as a control. Whole cell lysates were used as an input control. **C** 293 T cells were transfected with flag-WT-p53 or flag-R273h-p53 plasmids together with HA-P62 plasmids. The proteins were immunoprecipitated from cell extracts using anti-Flag antibody, followed by immunoblotting with anti-P62 antibodies. **D** 293 T cells were transfected with plasmids encoding wild-type p62 (HA-p62 wild type) or a p62 deletion mutant. The proteins were immunoprecipitated from cell extracts using anti-HA antibody, followed by immunoblotting with anti-P62 and anti-P53 antibodies. **E** Representative immunoblots of p53 and ubiquitinated p53 in U87 cells when p62 was upregulated or downregulated. The proteins were immunoprecipitated from cell extracts using anti-His antibody, followed by immunoblotting with anti-P53 antibodies. **F** 293 T cells were transfected with plasmids encoding HA-p53, pcDNA3.1 or HA-P62 together with His-ubiquitin or its indicated mutants (His-K48R-Ub, His-K63R-Ub). The proteins were immunoprecipitated from cell extracts using anti-His antibody, followed by immunoblotting with anti-P53 antibodies. **G** 293 T cells were transfected with plasmids encoding HA-p53 and His-ubiquitin together with HA-P62 or its indicated mutants. The proteins were immunoprecipitated from cell extracts using anti-P53 antibody, followed by immunoblotting with anti-P62 and anti-Ubiquitin antibodies

In mechanism study, we firstly tend to explore the mechanism of p62 regulating p53. Previous study has identified that p53 does not bind to p62 directly but is transferred to p62 through association with TGase 2 [23]. Lee and his colleagues found that p53 co-localized with p62 during cadmium exposure and genetic knockdown of p62 downregulated p53, and the cargo adaptor p62 might deliver p53 to the autophagosome during autophagy [24]. It was reported that, under sunitinib treatment, p62 could directly bind to p53 for its autophagic degradation with the help of HMGB1 [25]. Interesting, in our study, p62 overexpression resulted in an increase of HMW (high molecular weight)-p53 expression, despite of p53 status (Fig. 2A). To investigate the mechanism through which p62 regulates p53, we used immunofluorescence and co-immunoprecipitation assays to verify the potential association between p62 and p53. We examined the subcellular localization of p62 and p53 in U87 and U251 cells. We found that p62 and p53 shares similar localization in cytoplasm in part (Fig. 3A and Additional file 2: Figure S2). And the result from co-immunoprecipitation indicates that p62 associates with p53 (Fig. 3B). Then we compared the association of wild-type p53 or mutant p53 with p62. We constructed p53 mutant plasmids with R273h mutation, which contains the same p53 mutation with U251 cells. Flag-WT-p53 (wild-type p53) or flag-R273h-p53 (mutant p53) plasmids and HA-P62 plasmids were co-transfected into 293 T cells followed by IP analysis. The results showed that both wild-type p53 and mutant p53 (R273h) associates with p62 (Fig. 3C). We also found that erastin attenuates the association between p62 and p53 (Additional file 3: Figure S3).

To explore which part of p62 associates with p53, a series of HA-tagged deletion mutants of p62 was constructed, transfected into 293 T cells, and tested for immunoprecipitation using an anti-HA-tag antibody and western blotting using an anti-p53 antibody. Deletion mutant of p62 (Δ230-440) totally abolished association with p53; and other three deletion mutants including p62 (Δ1-385), (Δ124-440), (Δ1-227, 245–440) retained the association with p53 (Fig. 3D). These results suggest that the region residues 124–227 of p62 is responsible for association with p53. And we investigated ubiquitination of p53 by immunoprecipitation of p53 from lysates of U87 cells co-transfected with plasmids expressing HA-P53 WT and His-Ub, with or without HA-P62 WT. The result showed that ubiquitination of p53 was decreased in the presence of p62 (Fig. 3E). The K48R ubiquitin mutant attenuates the effect of p62 overexpression on p53 ubiquitination, but the K63R mutant did not (Fig. 3F). We also found that deletion mutant of p62 (Δ230-440) abolished the ability of downregulating p53 ubiquitination comparing to WT-p62 and other deletion mutants of p62 (Fig. 3G). We speculated that p62 associated with p53, suppressed its ubiquitination and increased accumulation of HMW-p53, resulting in the p53-mediated SLC7A11 inhibition. However, this hypothesis need more verification and it could only explain the inhibition of SLC7A11 in p53 mutant GBM, but could not explain the increase of SLC7A11 in p53 WT GBM upon p62 overexpression. We believed that there are other regulatory mechanisms.

### P53-NRF2 association and p53-mediated suppression of NRF2 antioxidant activity are diversely regulated by p62 according to p53 status

Accumulating evidence indicates that p53 regulates NRF2 signaling and mutant p53 may interact with NRF2 differentially regulating the NRF2-mediated antioxidant response [14–16]. This regulation is complicated, controversial and context-depend. We speculated the crosstalk between p62, NRF2 and p53 might be responsible for the dual role of p62 in ferroptosis.

**Fig. 4:**
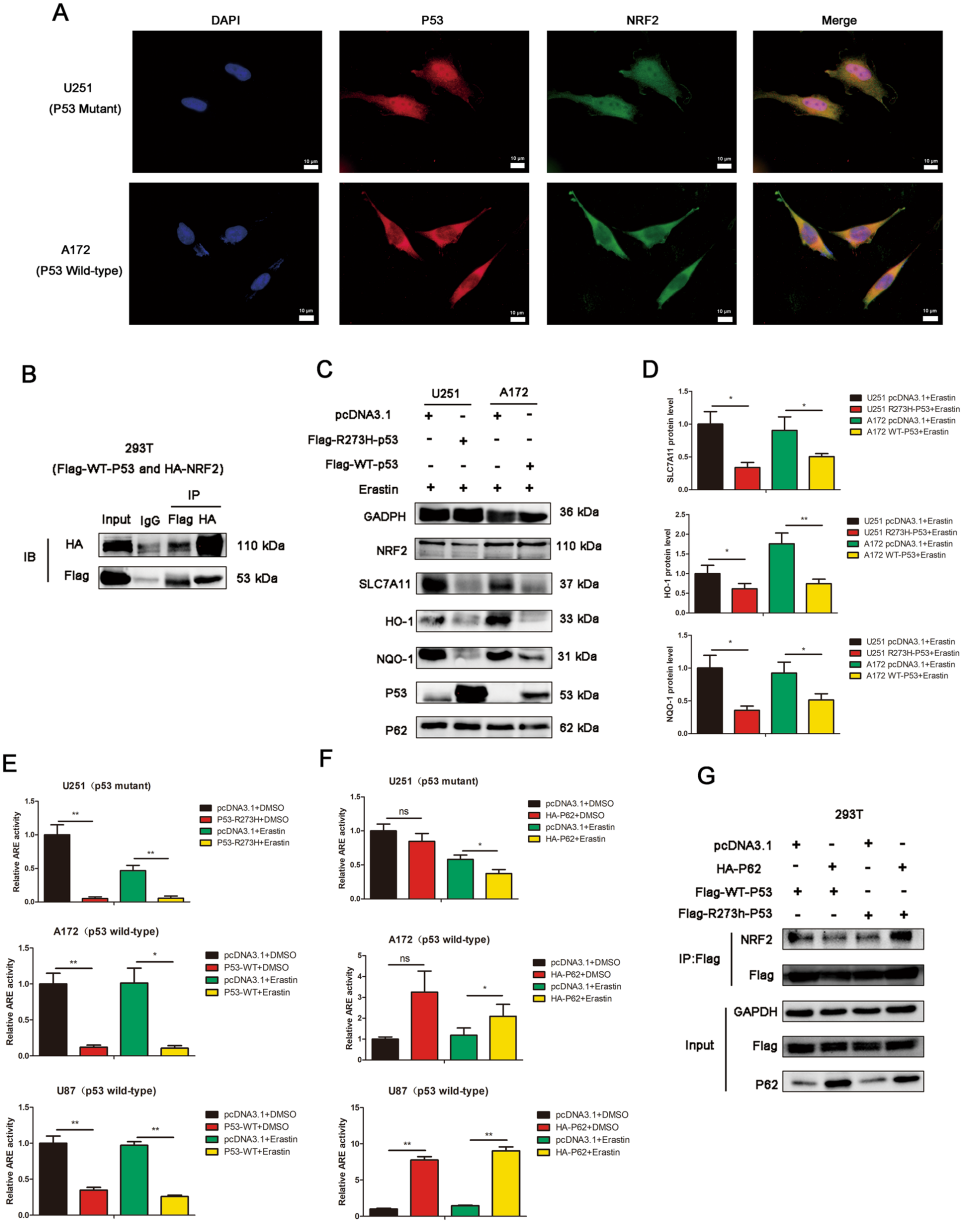
P53-NRF2 interaction and p53-mediated suppression of NRF2 activity are diversely regulated by p62 according to p53 status. **A** The localization of p53 and NRF2 was detected by immunofluorescence in U251 and A172 cells. Scar bars = 10 um. **B** Co-immunoprecipitation assays were performed to assess the association between NRF2 and p53. 293 T cells were co-transfected with HA-NRF2 and Flag-WT-p53 plasmids. Lysates of 293 T cells were subjected to IP using anti-Flag or anti-HA antibodies, followed by immunoblotting with anti-Flag and anti-HA antibodies. Non-specific IgG was used as a control. Whole cell lysates were used as an input control. **C** U251 cells were transfected with pcDNA3.1 or Flag-R273h-p53 plasmids, followed by erastin treatment or not. A172 cells were transfected with pcDNA3.1 or Flag-WT-p53 plasmids, followed by erastin treatment or not. The protein expression of SLC7A11, NRF2, p53, p62, HO-1 and NOQ-1 were detected by western blot analysis. **D** The quantification in Fig. 4C. **E** The overexpression of R273h mutant p53 inhibited the NRF2-driven luciferase activity in U251 cells by dual luciferase reporter assays. The overexpression of wild-type p53 inhibited the NRF2-driven luciferase activity in A172 and U87 cells by dual luciferase reporter assays. Cells were treated with DMSO or Erastin. The luciferase activity was calculated as Firefly luciferase/Renilla luciferase. **F** The effect of p62 overexpression on NRF2-driven luciferase activity in U251, U87 and A172 cells were determined by dual luciferase reporter assays. Cells were treated with DMSO or Erastin. **G** The effect of p62 overexpression on p53-NRF2 association in different p53 status was determined by IP analysis

We examined the sub-localization of p53 and NRF2 in U251 (p53 mutant), A172 (p53 wild-type) and U87 (p53 wild-type) cells by immunofluorescence. Partial co-localization of p53 with NRF2 was observed in U251 (p53 mutant), A172 (p53 wild-type) and U87 (p53 wild-type) cells (Fig. 4A and Additional file 4: Figure S4). Co-immunoprecipitation re-confirmed the association between p53 and NRF2 (Fig. 4B), which was reported by other researchers. NQO-1 and HO-1 are both downstream targets of NRF2. Western blots were performed to determine effect of wild-type p53 and mutant p53 on SLC7A11, NQO-1 and HO-1. Overexpression of mutant p53 decreased the expression of SLC7A11, NQO-1 and HO-1 in U251 (p53 mutant) cells upon erastin treatment, and overexpression of wild-type p53 decreased the expression of SLC7A11, NQO-1 and HO-1 in A172 (p53 wild-type) cells upon erastin treatment (Fig. 4C-D). By luciferase assay, we examined the impact of p53 on the activity of NRF2 signaling pathway. Overexpression of R273h mutant p53 inhibited the activity of NRF2 signaling pathway in U251 (p53 mutant) cells, and overexpression of wild-type p53 inhibited the activity of NRF2 signaling pathway in A172 (p53 wild-type) cells and U87 (p53 wild-type) cells (Fig. 4E). In 293 T cells, overexpression of wild-type or R273h mutant p53, could reduced the activity of NRF2 signaling pathway (Additional file 5: Figure S5). These results indicated that p53 could associate with NRF2 and inhibit the activity of NRF2 signaling pathway and lead to the decrease of SLC7A11, HO-1 and NQO-1, despite of p53 status.

The effect of p62 overexpression on the activity of NRF2 signaling pathway were also determined. In U87 (p53 wild-type) and A172 (p53 wild-type) cells, p62 overexpression enhanced the activity of NRF2 signaling pathway in the existence of erastin (Fig. 4F), which is consistent with the classical p62-NRF2 pathway. Similar results was also confirmed in 293 T cells (Additional file 6: Figure S6). However, in U251 (p53 mutant) cells, p62 overexpression reduced the activity of NRF2 signaling pathway in the existence of erastin. Since p62/NRF2 activation is the classical pathway, there are other mechanisms that lead to the discrepancy.

Then we determined whether p62 affects the p53-NRF2 association. The results showed that p62 overexpression could enhance the association between NRF2 and R273h mutant p53, but had no significant effect on the association between NRF2 and wild-type p53 (Fig. 4G). We conjectured that the enhanced mutant-p53/NRF2 association by p62 lead to stronger inhibition of NRF2 antioxidant activity. It is speculated that p53-NRF2 association and p53-mediated suppression of NRF2 antioxidant activity are diversely regulated by p62 according to p53 status, resulting to the dual role of p62 in GBM ferroptosis.

Interestingly, IP experiments with endogenous p53 and NRF2 were performed in U87 control cells and U87 CRISPR-P62 cells (Additional file 7: Figure S7). The results indicated that p53 and NRF2 still have association in p62-KO cells. It reminds us that the association of P53 and NRF2 is not totally dependent on p62, at least in U87 (p53 WT) cells.

### P53 mutation status is responsible for the dual regulation of p62 on ferroptosis

**Fig. 5:**
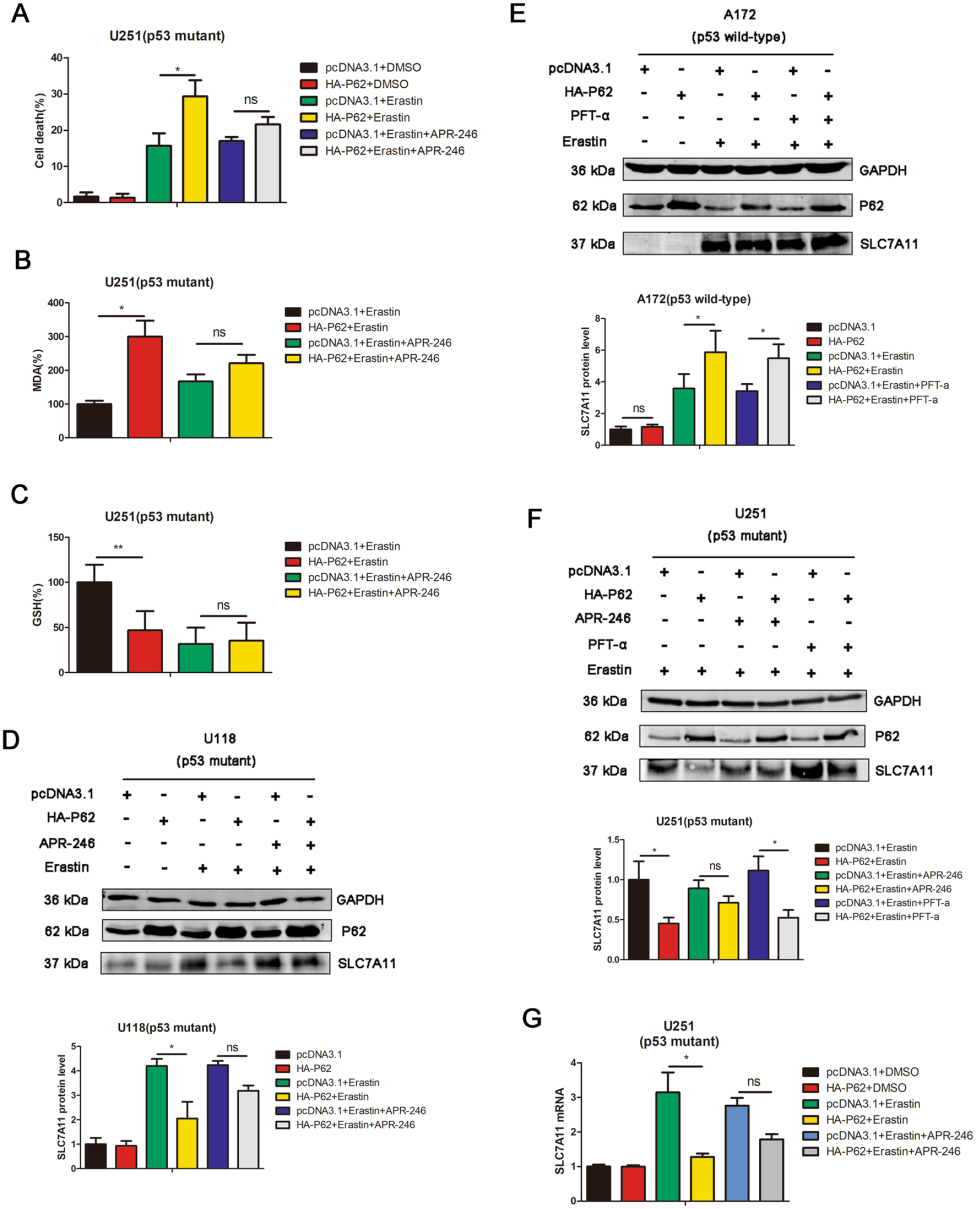
P53 mutation status is responsible for the dual regulation of p62 on GBM ferroptosis. **A** Cells were transfected with pcDNA3.1 or HA-P62 plasmids, followed by DMSO, erastin or erastin + APR-246 treatment. Cell death were determined by trypan blue staining. **B** The levels of MDA were measured in U251 cells, which were transfected with pcDNA3.1 or HA-P62 plasmids, followed by erastin or erastin + APR-246 treatment. **C** The levels of GSH were measured in U251 cells, which were transfected with pcDNA3.1 or HA-P62 plasmids, followed by erastin or erastin + APR-246 treatment. **D** U118 cells were transfected with pcDNA3.1 or HA-P62 plasmids, followed by DMSO or erastin or erastin + APR-246 treatment. The protein expression of SLC7A11 and P62 were detected by western blot analysis. **E** A172 cells were transfected with pcDNA3.1 or HA-P62 plasmids, followed by DMSO or erastin or erastin + PFT-a treatment. The protein expression of SLC7A11 and P62 were detected by western blot analysis. **F** U251 cells were transfected with pcDNA3.1 or HA-P62 plasmids, followed by DMSO or erastin or erastin + APR-246 treatment. The protein expression of SLC7A11 and P62 were detected by western blot analysis. **G** U251 cells were transfected with pcDNA3.1 or HA-P62 plasmids, followed by DMSO or erastin or erastin + APR-246 treatment. The mRNA expression of SLC7A11 were detected by qPCR

APR-246 is a small-molecule drug that reactivates mutant p53, thereby restoring wild-type p53 function [26]. To further verify the hypothesis that p53 and its mutation status are required for the dual regulation of GBM ferroptosis mediated by p62, we employed APR246 to perform the rescue experiment. As shown in Fig. 5A–C APR-246 reversed p62-mediated ferroptosis enhancement in U251 (p53 mutant) cells. The result from western blotting also indicated that APR-246 reversed the decrease of SLC7A11 expression mediated by p62 overexpression in U118 (p53 mutant) cells (Fig. 5D). As for p53 wild-type GBM cells, we used pifithrin-α (p53 inhibitor) to perform the rescue experiment, but the results indicated that pifithrin-α (PFT-a) could not significantly affect the impact of p62 overexpression on SLC7A11 (Fig. 5E) in A172 (p53 wild-type) cells. We also compare the effect of APR-246 and pifithrin-α in U251 (p53 mutant) cells. APR-246, but not pifithrin-α, could reverse the effect of p62 overexpression on SLC7A11 in U251 (p53 mutant) cells (Fig. 5F). By real-time PCR, APR-246 reversed the decrease of SLC7A11 mRNA expression mediated by p62 overexpression in U251 (p53 mutant) cells (Fig. 5G).

All these results revealed that APR-246 reversed the effect of p62 overpression on ferroptosis in p53 mutant GBM, indicating that p53 mutation status is required for the dual regulation of GBM ferroptosis mediated by p62.

### The clinical expression pattern and survival analysis of p62 in p53-wild-type and p53-mutant gliomas

**Fig. 6:**
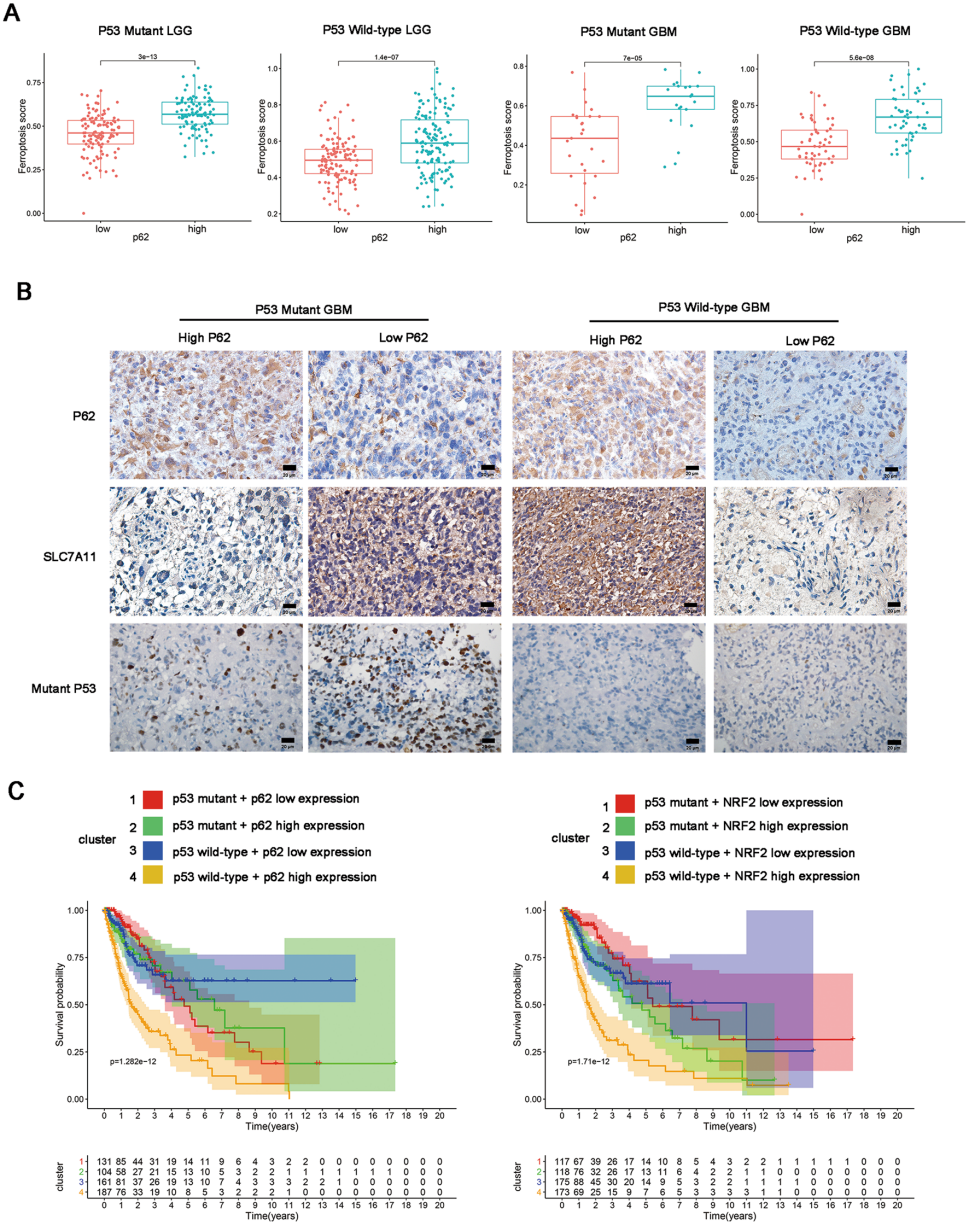
The clinical expression pattern and survival analysis of p62 in p53-wild-type and p53-mutant gliomas. **A** Difference analysis of ferroptosis enrichment score (ES) among different subtypes. According to the median value of p62, the level of ES were compared among low and high expression group in p53 mutant LGG, p53 wild-type LGG, p53 mutant GBM and p53 wild-type GBM. **B** Representatives of IHC staining of p62 and SLC7A11 expression in p53 mutant GBM and p53 wild-type GBM tissues. **C** Survival analysis of OS in four clusters in p62 (left) and NRF2 (right) respectively. Cluster 1: p53 mutant + p62 or NRF2 low expression, cluster 2: p53 mutant + p62 or NRF2 high expression, cluster 3: p53 wild-type + p62 or NRF2 low expression, cluster 4: p53 wild-type + p62 or NRF2 high expression

To verify the role p62 of GBM ferroptosis from clinical sample levels, we performed bioinformatic analysis and IHC staining. 587 tumor samples of TCGA-glioma dataset were divided into four subtypes: p53-mutant LGG, p53-Wild-type LGG, p53-mutant GBM and p53-Wild-type GBM, according to the status of p53 mutation. In each subtype, samples were divided into low or high expression group by the median value of p62 or NRF2. The gene set “WP_Ferroptosis” representing the activity of ferroptosis pathways was obtained from Molecular Signatures Database and was quantified for their enrichment degrees (enrichment score, ES) within respective in each glioma samples. We found that p62 high expression groups had higher levels of Ferroptosis Score than p62 low expression group in p53-mutant LGG, p53-Wild-type LGG, p53-mutant GBM and p53-Wild-type GBM (Fig. 6A). NRF2 high expression groups had higher levels of Ferroptosis Score than NRF2 low expression group in p53-mutant LGG, p53-Wild-type LGG, p53-mutant GBM and p53-Wild-type GBM (Additional file 8: Figure S8). This result indicates that p62 and NRF2 are highly associated with ferroptosis in clinical sample levels. IHC staining showed that p62 expression has a positive correlation with SLC7A11 in p53-wild-type GBM, but has an inverse correlation with SLC7A11 in p53-mutant GBM (Fig. 6B).

We also performed survival analysis using bioinformatic data. According to p53 mutation status and the median of p62 or NRF2 respectively, patients with glioma were divided into p53 mutant + p62/NRF2 low expression, p53 wild-type + p62/NRF2 low expression, p53 mutant + p62/NRF2 high expression and p53 wild-type + p62/NRF2 high expression group. The results indicates that p53 wild-type + p62/NRF2 high expression group has the poorest outcome than other groups (Fig. 6C).

### Depletion of p62 promotes ferroptosis in p53 wild-type GBM with an intracranial xenograft model

**Fig. 7:**
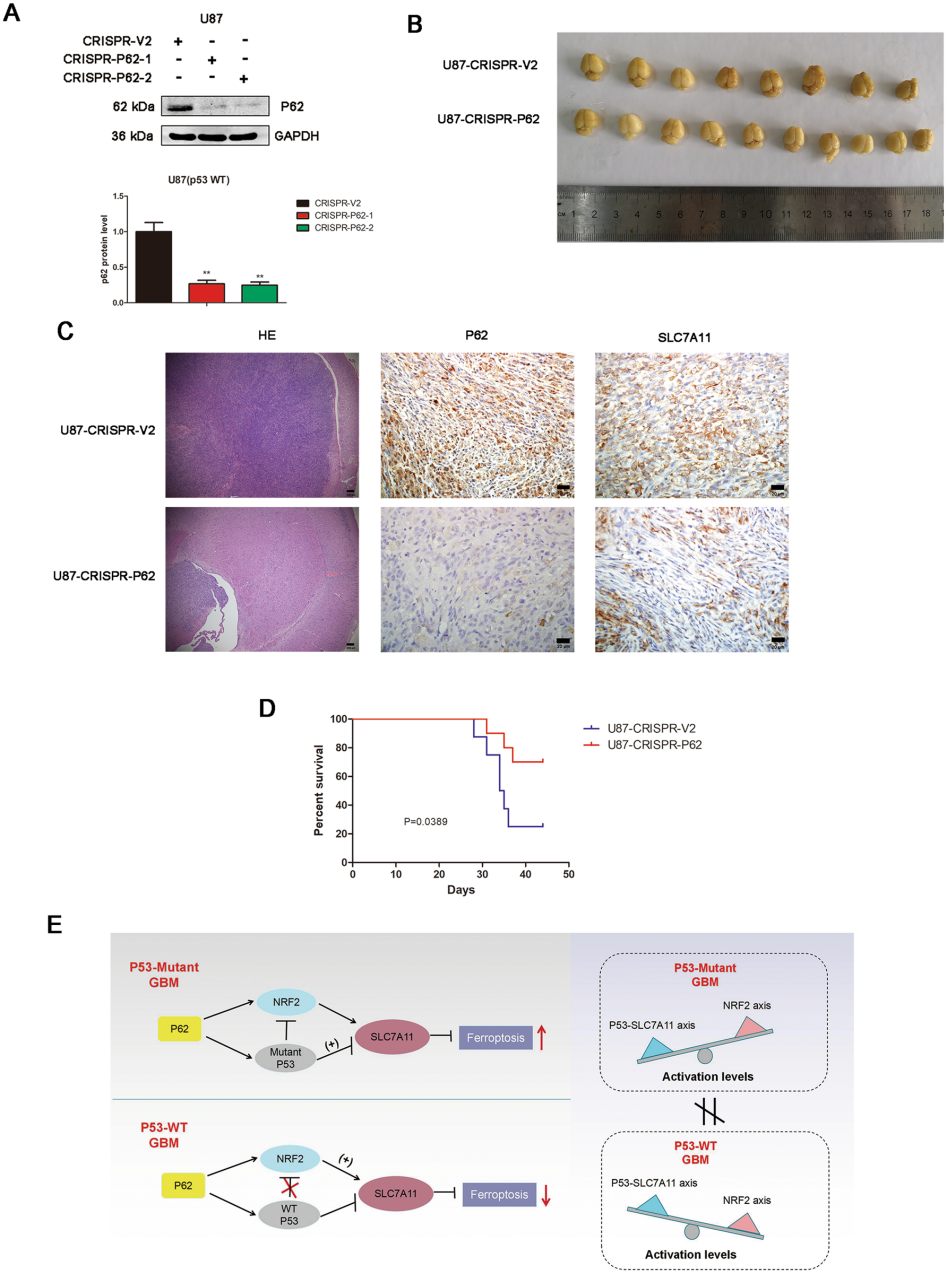
Depletion of p62 promotes ferroptosis in p53 wild-type GBM with an intracranial xenograft model. **A** The CRISPR/CAS9 system was applied to construct stable p62 knockout U87 cells (CRISPR-p62-1 and CRISPR-p62-2). Knockout efficacy was verified by western blot analysis. **B** Representative images of pLenti-CRISPR-V2 (CTRL) (n = 8) and pLenti-CRISPR-p62-1 (CRISPR-p62) (n = 10) cells from mouse brains. **C** H&E and IHC analyses of p62 and SLC7A11 in orthotopic tumour sections. **D** Mouse survival is shown by Kaplan–Meier curves. U87-CRISPR-V2 groups, n = 8. U87-CRISPR-P62 groups, n = 10. P values were calculated using the log-rank test. **E** Mechanistic model for dual role of p62 in ferroptosis according to p53 status in GBM

To verify the role of p62 on ferroptosis in vivo, the intracranial xenograft model were constructed. The knockout efficacy was validated by western blot (Fig. 7A). U87 (p53 wild-type) cells developed larger tumours than the mice implanted with the.

p62 knockout cells (CRISPR-p62) (Fig. 7B). IHC staining indicated that p62 depletion caused a decrease of SLC7A11 expression (Fig. 7C). Survival analysis revealed that the p62 knockout group mice survived significantly longer than the control group (Fig. 7D).

Since P62 is a well-known receptor for autophagy, we also examined the expression of autophagy markers including ATG7, Beclin-1 and LC3B in xenograft tumor sections by IHC. However, the result showed that there was no significant change of ATG7, Beclin-1 and LC3B expression after p62 depletion (Additional file 9: Figure S9).

## Discussion

P62, mainly defined as a cargo receptor for selective autophagy, also interacts with various signaling proteins to regulate a variety of cellular functions. Recent evidence indicates that p62 acts as a potential regulator for ferroptosis. Sun et al. found that activation of the p62-Keap1-NRF2 pathway protects against ferroptosis in hepatocellular carcinoma cells [26] and protects 6-Hydroxydopamine-induced ferroptosis in dopaminergic cells [27]. However, little is known about the function of p62 in glioma cell ferroptosis. In this study, we found that p62 promotes ferroptosis in p53-mutant GBM cells but inhibits ferroptosis in p53-wild-type GBM cells. The opposite effect of p62 on ferroptosis in different p53 mutation status excited our interest and we performed further study to reveal the mechanism.

We examined the impact of p62 on SLC7A11, which is a key ferroptosis marker, and the results indicated that p62 overexpression increased the level of SLC7A11 in p53 WT GBM cells but decreased the level of SLC7A11 in p53 mutant GBM cells. Although the activation of classical p62-NRF2 signaling increased the expression of SLC7A11 to protect against ferroptosis in hepatocellular carcinoma cells [10], but this theory alone could not explain the opposite effect of p62 on ferroptosis in different p53 mutation status. It is well accepted that SLC7A11, which is involved in the formation of system XC -, is recognized as a key factor of anti-ferroptosis [10]. The promoter region of SLC7A11 contains a p53 binding site, and p53 can reduce the activity of system XC—by inhibiting the transcription of SLC7A11 to promote ferroptosis [12]. Xie et al. found that p53 limits ferroptosis by blocking DPP4 activity [13]. It seems that p53 has the potential of bidirectional regulation in ferroptosis, rather than the antioxidant function of classical p62-NRF2 signaling. Based on the above, we speculate that p53 and NRF2 signaling pathway may both be involved in p62-mediated ferroptosis regulation. Thus we tend to explore how p62 affects p53 and NRF2 signaling pathway.

One of the important topic of our research is the mechanism of p62 regulating p53. Accumulating studies have reported the relationship between p62 and p53, but the conclusion is still controversial, and the exact mechanism is not clear. Kang et al. found that p53 does not bind to p62 directly but is transferred to p62 through association with TGase 2, revealing a triple complex of p53-TGase2-p62 and proposing a mechanism of TGase 2-mediated chaperoning of p53 to the autophagosome [23]. It is reported that p53 co-localized with p62 during cadmium exposure and genetic knockdown of p62 downregulated p53, and the cargo adaptor p62 might deliver p53 to the autophagosome during autophagy [24]. Luo et al. claimed that, under sunitinib treatment, p62 could directly bind to p53 for its autophagic degradation with the help of HMGB1 [25]. Kalathil et al. performed interactome analysis and revealed an association of p53 with autophagosome complex associated proteins including BAG3, p62 and HSC70 under thiostrepton treatment [28]. We explore the relationship between p62 and p53 signaling pathway and the results indicate that p62 associates with p53, inhibits the ubiquitination of p53. Based on these results, we speculate that p62 activates p53 by associating with p53 and inhibiting its ubiquitination. However, there are several problems about the regulation of p62 on p53 to be solved. Firstly, the association between p62 and p53 is likely to be indirect. In other words, “p62 associates with p53” might be a more appropriate description than “p62 interacts with p53”. In our studies we did not confirm the chaperone protein between p62 and p53 and whether this chaperone protein mediates the inhibition of p53 ubiquitination? We will investigate it in our further study. Secondly, since p62 is the receptor protein for selective autophagy of ubiquitinated cargos, is it possible that autophagy involved in the regulation of our study? As previous literature described, p62 may deliver or interact with p53 for its autophagic degradation under certain stress [24, 25, 28]. Does p62 inhibit the ubiquitination of p53 in proteasome-dependent way or in autophagy-dependent pathway? Or there may be other unknown mechanisms. Thirdly, is the logical sequence of signaling pathways as we speculated that p62 promotes p53 signaling pathway by associating with p53 and inhibiting its ubiquitination? Lastly, in our study, we observed high molecule weight of p53 in cells overexpressed with p62, but the exact mechanism is not clear. Further studies are needed to explore the exact mechanism.

Ferroptosis is regulated by core transcription factors p53 and NRF2 through multiple mechanisms. Transactivation of NRF2 target antioxidant genes (such as SLC7A11, HO-1 and NQO-1) mediates decrease of lipid peroxides and inhibits ferroptosis [10, 29–31]. p53 has been reported to regulate ferroptosis both positively and negatively [12, 13, 32]. It is reported that the activation of p53 induced by nutlin-3 can inhibit the expression of SLC7A11 [3]. Jiang et al. found that p53 can reduce the activity of system XC—by inhibiting the transcription of SLC7A11, and promote the ferroptosis of osteosarcoma U2OS cells and breast cancer MCF7 cells [12]. Also, p53-dependent regulation of dipeptidyl-peptidase-4 (DPP4) [13] and p21 [32] has been shown to inhibit ferroptosis. But the crosstalk between p53 and NRF2 in ferroptosis remains unclear, especially in different p53 mutation status.

The regulation of p53 on NRF2 is interesting and controversial. Faraonio et al. found that p53 suppressed the NRF2-dependent transcription of antioxidant response genes through binding to promoter elements activated by NRF2 [16]. However, three p53 targets, SESN1/2 [33] and p21 [34], have been reported to interfere with Keap1-NRF2 complex, thus enhancing NRF2 stability and activity. Kalo et al. found that wild-type p53 promotes the induction of NRF2 target genes under oxidative stress [35]; but mutant P53 (R273H) attenuates the activation and antioxidant response of NRF2, leading to decreased expression of NRF2 target genes such as HO-1 and NQO-1 [35]. It is reported that mutant p53 suppresses the NRF2-dependent transcription of SLC7A11 through binding to NRF2 [36]. In another study it was shown that mutant p53 binding to NRF2 leads to an increased nuclear localization of NRF2 on ARE-containing regulatory sequences, resulting in the transcriptional activation of genes such as TXN and the proteasome encoding genes, and in the repression of others such as HO-1, SLC7A11 and ABCC3 [14]. Another study indicates that NRF2 interacts with p53 mutants in breast cancer cells [37]; and APR-246, a first-in-class mutant p53 reactivator, disrupts the interaction between mutant P53 and NRF2 [37]. But in non-small cell lung cancer, it was reported that NRF2 promoter activity and its mRNA levels were markedly suppressed by wild-type p53, but not by mutant p53; while mutant p53 could upregulate NRF2 expression at the transcriptional level, thereby conferring cisplatin resistance [15]. Gilardini et al. found that mutant p53 activated NRF2 to promote p62 transcription and that p62 in turn contributed to NRF2-mediated activation of antioxidant response in PaCa44 cells [38]. None of the above studies was performed in glioma. Some scientists believe that while low levels of p53 can induce NRF2, conditions that lead to high p53 protein levels seem to compromise NRF2 activity [39]. The discrepancies and controversy between these researches indicate that the crosstalk between NRF2 and p53 might be dependent on the cellular and biological context.

The role of p62 on p53-NRF2 assocation is not fully explored yet. Our study indicated that p53 and NRF2 have association both in U87 p62 control and U87 p62-KO cells (Additional file 7: Figure S7). It reminds us that the association of P53 and NRF2 is not totally dependent on p62, at least in U87 (p53 WT) cells. But exogenous p62 overexpression could lead to enhanced mutant-p53/NRF2 association (Fig. 4G). The regulator of p53-NRF2 interaction is complicated and not fully clarified. It may include the level of p62, the status of p53, cell conditions, and et al. The exact regulatory mechanism of p53-NRF2 association still need further studies.

The accumulation of HMW (high molecular weight)-p53 in p62 overexpression group were observed in this study. The definition of HMW-p53 contains various possibilities. Since p53 post-translational modifications play important role in its biological function, HMW-P53 may be a form of post-translational modified p53, including ubiquitination, phosphorylation, etc. Of course, it could be just nonspecific binding by the antibody. The function of HMW-p53 is not clear and it includes many interesting questions. What is the function of HMW-P53 in regulating ferroptosis? Will the two different mutations (p53 R273H and R213Q) affect p53 ubiquitination or the ratio of HMW-P53 and p53? Is the function of HMW-p53 and p53 the same or different in ferroptosis? An in-depth understanding of HMW-p53 is an interesting topic and need further studies.

Our study indicates p53 could associate with NRF2 and inhibit the antioxidant activity of NRF2 signaling pathway. P62 overexpression could enhance the association between NRF2 and R273h mutant p53, but could not significantly affect the association between NRF2 and wild-type p53. P53-NRF2 association and p53-mediated suppression of NRF2 antioxidant activity are diversely regulated by p62 according to p53 status. We conjectured that the enhanced mutant-p53/NRF2 association by p62 lead to stronger inhibition of NRF2 antioxidant activity in p53 mutant GBM. Considering our results that p62 associates with p53 and the classical p62-mediated NRF2 activation pathway, we speculate that the diverse crosstalk of p53/NRF2 might be responsible for the dual function of p62 in GBM ferroptosis according to p53 status.

We propose the mechanistic model for dual role of p62 in ferroptosis according to p53 status in GBM (Fig. 7E). P62 associates with p53, inhibits its ubiquitination and enhance p53-mediated transcriptional suppression on SLC7A11. P53-NRF2 association and p53-mediated suppression of NRF2 antioxidant activity are diversely regulated by p62 according to p53 status. In wild-type p53 GBM, the classical p62-mediated NRF2 activation pathway plays a major regulatory role of ferroptosis, leading to increased SLC7A11 expression, resulting in a anti-ferroptosis role. In mutant p53 GBM, stronger interaction of mutant-p53/NRF2 by p62 enhance the inhibitory effect of mutant p53 on NRF2 signaling, which reversing the classical p62-mediated NRF2 activation pathway, together with increased p53’s transcriptional suppression on SLC7A11 by p62, leading to a decrease of SLC7A11, resulting in a pro-ferroptosis role.

However, our mechanistic model still needs further elucidation. Firstly, the exact mechanism that p62 affects mutant-p53/NRF2 association remains unclear. And the hypothesis that p53/NRF2 association lead to the inhibition of NRF2 signaling pathway still need more verification. Secondly, there are many different mutations in p53 and does the dual function of p62 on ferroptosis suits for other kinds of p53 mutation? Thirdly, is it possible that NRF2 affects p53 signaling in a feedback loop mediated by p62? Previous findings from the Shaul lab indicate that NQO1, an NRF2 target, interacts with p53 [40] and blocks its degradation by the 20S proteasome [41]. A possibility exists that the feedback loop of p62/p53/NRF2 plays an important role in the regulation of ferroptosis in glioma according to p53 mutation status. Lastly, since p62 plays an key role in autophagy and an increasing number of researches have reported significant crosstalk between ferroptosis and autophagy, we could not exclude the possibility that p62-related autophagy take part in ferroptosis regulation. P62 might be a bridge between autophagy and ferroptosis, although we did not explore it in depth in this study. All these unsolved and interesting problems need our further studies.

p53 has acquired a central fame in cancer research and one reason for that is the frequent mutation of the TP53 gene in human tumors. About 30%—50% of GBM contain p53 mutation, and these patients with p53 mutant GBM have poor chemosensitivity and worse prognosis than those with wild-type p53 GBM [42, 43]. Our studies indicate that the dysregulation of ferroptosis might be the reason for death escape and malignant progression of GBM which contain high levels of p53 mutation. And molecular therapies against p62/p53/NRF2 combined with APR-246 might have potential clinical value for comprehensive treatment of GBM.

## Conclusions

This study shows novel molecular mechanisms of ferroptosis regulated by p62; the mutation status of p53 is an important factor that determines the therapeutic response to p62-mediated ferroptosis-targeted therapies in GBM.

## Supplementary Information

**Additional file 1: Figure S1.** Endogenous expression of SLC7A11, p53, p62 and NRF2 in GBM cells. Endogenous expression of SLC7A11, p53, p62 and NRF2 were determined by western blot in U251, U118, U87 and A172 cells under normal conditions.

**Additional file 2: Figure S2.** The localization of p62 and p53 in U251 cells. The localization of p62 and p53 was detected by immunofluorescence in U251 cells. Scar bars = 10 um.

**Additional file 3: Figure S3.** The effect of erastin on the association of p62 and p53. Immunoprecipitation was used to assess the effect of erastin on the association between p62 and p53. Lysates of U87 cells were subjected to IP using anti-P62 antibodies, followed by immunoblotting with anti-P62 and anti-P53 antibodies.

**Additional file 4: Figure S4.** The localization of p53 and NRF2 in U87 cells. The localization of p53 and NRF2 was detected by immunofluorescence in U87 cells. Scar bars = 10 um.

**Additional file 5: Figure S5.** The impact of wild-type or mutant p53 on the activity of NRF2 signaling pathway in 293 T cells. The overexpression of wild-type or R273h mutant p53 decreased the p53-driven luciferase activity in 293 T cells by dual luciferase reporter assays. Cells were treated with DMSO or Erastin. The luciferase activity was calculated as Firefly luciferase/Renilla luciferase.

**Additional file 6: Figure S6.** The impact of p62 on the activity of NRF2 signaling pathway in 293 T cells. The overexpression of p62 increased the NRF2-driven luciferase activity in 293 T cells by dual luciferase reporter assays. Cells were treated with DMSO or Erastin. The luciferase activity was calculated as Firefly luciferase/Renilla luciferase.

**Additional file 7: Figure S7.** P53 and NRF2 have association both in U87 control cells and U87 P62-KO cells. Lysates of U87 control cells (CRISPR-V2) and U87 p62-KO cells (CRISPR-P62) were subjected to IP using anti-p53 antibodies, followed by immunoblotting with anti-NRF2 and anti-p53 antibodies. Whole cell lysates were used as an input control.

**Additional file 8: Figure S8.** Difference analysis of ferroptosis enrichment score (ES) according to NRF2. Difference analysis of ferroptosis enrichment score (ES) among different subtypes. According to the median value of NRF2, the level of ES were compared among low and high expression group in p53 mutant LGG, p53 wild-type LGG, p53 mutant GBM and p53 wild-type GBM.

**Additional file 9: Figure S9.** The impact of p62 depletion on autophagy markers in intracranial xenograft model. IHC staining were performed to detect the expression of ATG7, Beclin-1 and LC3B in orthotopic tumour sections. Scar bars = 20 um.

## Data Availability

Not applicable.

## Abbreviations

GBM: Glioblastoma
LGG: Low grade glioma
TEM: Transmission electron microscopy
ROS: Reactive oxygen species
IP: Immunoprecipitation
