# Supplementary figures and images for: The dual role of p62 in ferroptosis of glioblastoma according to p53 status
Source: Cell Biosci. 2022 Feb 25;12:20. doi: 10.1186/s13578-022-00764-z (PMC8881833; doi:10.1186/s13578-022-00764-z)

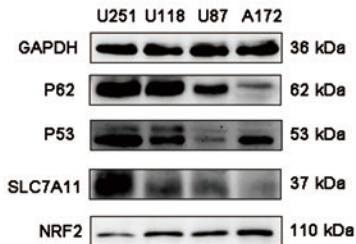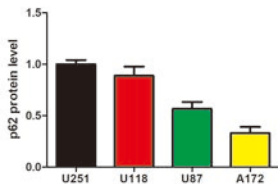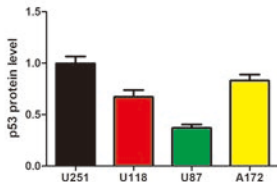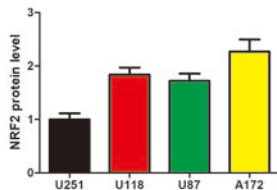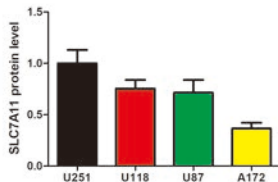

Supplement: Supplementary file 1 — Additional file 1: Figure S1. Endogenous expression of SLC7A11, p53, p62 and NRF2 in GBM cells. Endogenous expression of SLC7A11, p53, p62 and NRF2 were determined by western blot in U251, U118, U87 and A172 cells under normal conditions. [file 13578_2022_764_MOESM1_ESM.pdf]

U251  
(P53 mutant)

DAPI

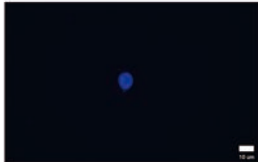

P53

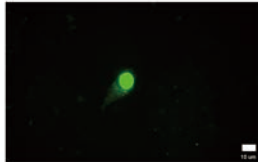

P62

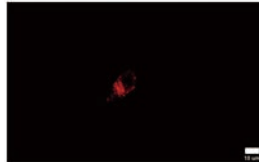

Merge

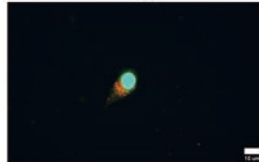

Supplement: Supplementary file 2 — Additional file 2: Figure S2. The localization of p62 and p53 in U251 cells. The localization of p62 and p53 was detected by immunofluorescence in U251 cells. Scar bars = 10 um. [file 13578_2022_764_MOESM2_ESM.pdf]

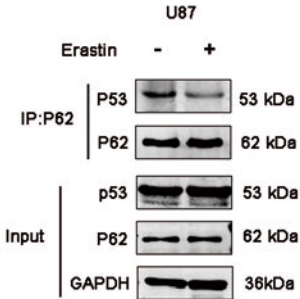

Supplement: Supplementary file 3 — Additional file 3: Figure S3. The effect of erastin on the association of p62 and p53. Immunoprecipitation was used to assess the effect of erastin on the association between p62 and p53. Lysates of U87 cells were subjected to IP using anti-P62 antibodies, followed by immunoblotting with anti-P62 and anti-P53 antibodies. [file 13578_2022_764_MOESM3_ESM.pdf]

U87  
(P53 Wild-type)

DAPI

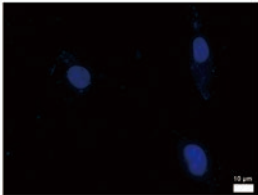

P53

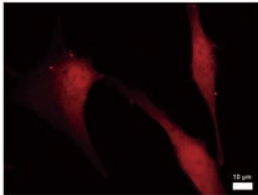

NRF2

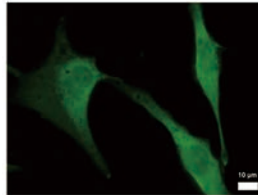

Merge

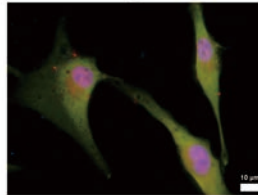

Supplement: Supplementary file 4 — Additional file 4: Figure S4. The localization of p53 and NRF2 in U87 cells. The localization of p53 and NRF2 was detected by immunofluorescence in U87 cells. Scar bars = 10 um. [file 13578_2022_764_MOESM4_ESM.pdf]

## 293T

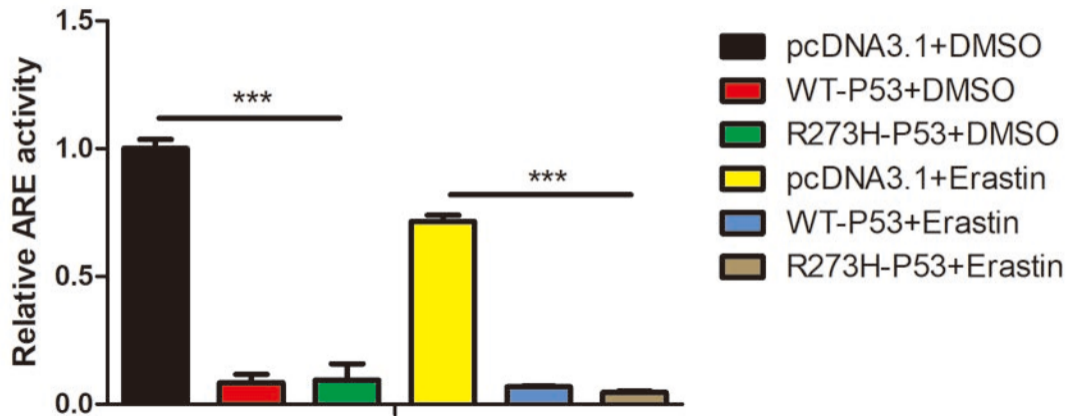

Supplement: Supplementary file 5 — Additional file 5: Figure S5. The impact of wild-type or mutant p53 on the activity of NRF2 signaling pathway in 293 T cells. The overexpression of wild-type or R273h mutant p53 decreased the p53-driven luciferase activity in 293 T cells by dual luciferase reporter assays. Cells were treated with DMSO or Erastin. The luciferase activity was calculated as Firefly luciferase/Renilla luciferase. [file 13578_2022_764_MOESM5_ESM.pdf]

293T

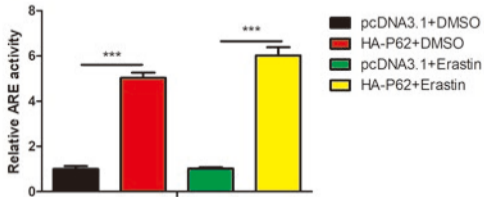

Supplement: Supplementary file 6 — Additional file 6: Figure S6. The impact of p62 on the activity of NRF2 signaling pathway in 293 T cells. The overexpression of p62 increased the NRF2-driven luciferase activity in 293 T cells by dual luciferase reporter assays. Cells were treated with DMSO or Erastin. The luciferase activity was calculated as Firefly luciferase/Renilla luciferase. [file 13578_2022_764_MOESM6_ESM.pdf]

543

CR0PS-P3

+

+

CR0PS-P4

-

+

IP-P43

NP1

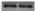

110 kHz

P43

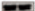

53 kHz

Input

SAPO4

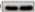

38 kHz

P55

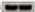

55 kHz

P43

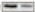

63 kHz

NP1

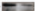

110 kHz

Supplement: Supplementary file 7 — Additional file 7: Figure S7. P53 and NRF2 have association both in U87 control cells and U87 P62-KO cells. Lysates of U87 control cells (CRISPR-V2) and U87 p62-KO cells (CRISPR-P62) were subjected to IP using anti-p53 antibodies, followed by immunoblotting with anti-NRF2 and anti-p53 antibodies. Whole cell lysates were used as an input control. [file 13578_2022_764_MOESM7_ESM.pdf]

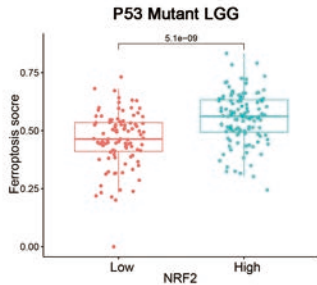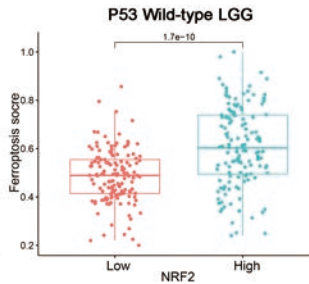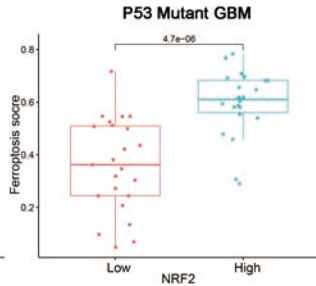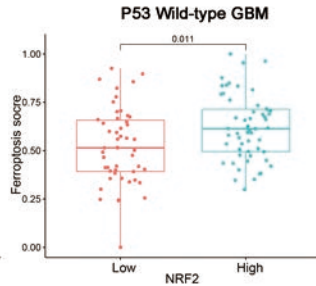

Supplement: Supplementary file 8 — Additional file 8: Figure S8. Difference analysis of ferroptosis enrichment score (ES) according to NRF2. Difference analysis of ferroptosis enrichment score (ES) among different subtypes. According to the median value of NRF2, the level of ES were compared among low and high expression group in p53 mutant LGG, p53 wild-type LGG, p53 mutant GBM and p53 wild-type GBM. [file 13578_2022_764_MOESM8_ESM.pdf]

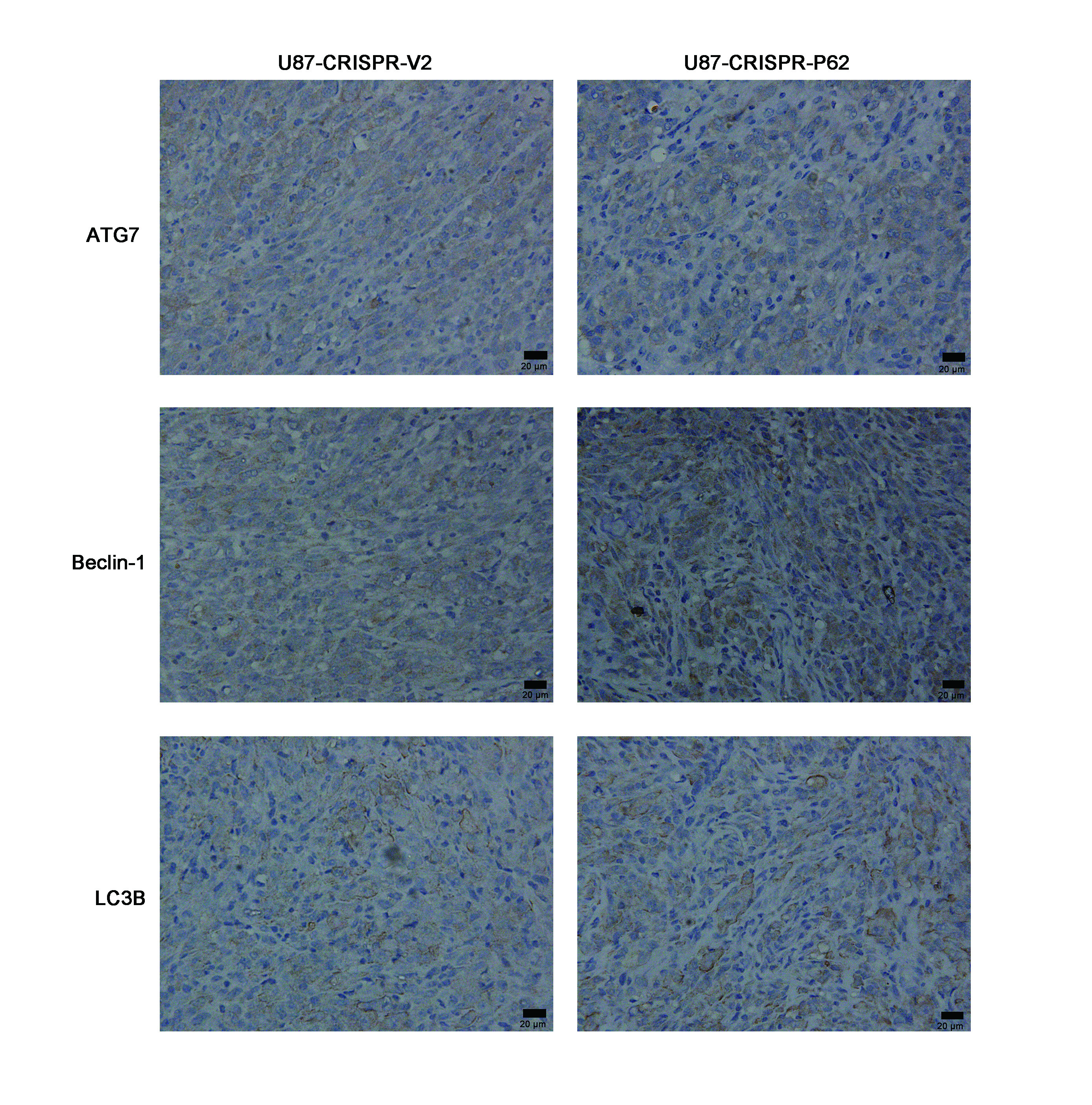

Supplement: Supplementary file 9 — Additional file 9: Figure S9. The impact of p62 depletion on autophagy markers in intracranial xenograft model. IHC staining were performed to detect the expression of ATG7, Beclin-1 and LC3B in orthotopic tumour sections. Scar bars = 20 um. [file 13578_2022_764_MOESM9_ESM.jpg]
